# Using OpenPrescribing.net to evaluate neighbourhood-level prescribing of inhalers for asthma and COPD

**DOI:** 10.1038/s41598-025-02969-x

**Published:** 2025-05-24

**Authors:** Thomas C. Richards, Alison Heppenstall, Rachel A. Oldroyd, Victoria Barr, Roger Beecham

**Affiliations:** 1 University of Exeter, NIHR Exeter Biomedical Research Centre, Exeter, EX1 2LU UK; 2 University of Glasgow, School of Social and Political Science, Glasgow, G12 8RT UK; 3 University of Leeds, Consumer Data Research Centre, Leeds, LS2 9JT UK; 4 University of Leeds, School of Geography, Leeds, LS2 9JT UK; 5 Menston and Guiseley Surgery, Leeds, LS29 6HT UK; 6 University of Leeds, Leeds Institute for Data Analytics, Leeds, LS2 9JT UK

**Keywords:** OpenPrescribing, Inhalers, Asthma, COPD, Health inequalities, Prescribing safety, Health care, Health care economics, Health policy, Health services, Public health, Respiration, Environmental impact, Risk factors

## Abstract

Prescribing of inhalers for asthma and chronic obstructive pulmonary disease is complicated due to multiple treatment pathways, diverse products, and variability in patients’ needs and usage habits. Factors such as social deprivation, air quality, and differences in access to primary care influence both demand on respiratory medications and the rate and manner in which they are prescribed. Inhaler prescribing metrics are valuable for analysing temporal and geographic prescribing patterns across socio-economic groups, whether to identify areas with higher disease incidence or to assess problematic prescribing practices. Using data from OpenPrescribing.net, we estimate prescription items dispensed for different inhaler drugs in England at Lower Layer Super Output Area or ‘neighbourhood’ level. Generating metrics at small-area level enables analysis of the interactions between individuals, their environment, and the localised health system within which they are being treated. This approach complements a patient-centred clinical model that considers individual patient needs. To explore changes pre- and post- the March 2020 COVID-19 lockdown, we profile prescribing temporally and by neighbourhood-level deprivation, measured by the Index of Multiple Deprivation. We develop and apply seven prescribing measures informed by national clinical guidelines which facilitate contextual comparison of prescribing behaviour between neighbourhoods. We find inequalities in prescribing behaviour, with the most deprived neighbourhoods associated with higher rates of emergency or ’rescue’ inhaler prescribing - a pattern that was interrupted during the March 2020 lockdown. Additionally, we find an increase in the prescribing of less environmentally friendly inhalers, contrary to national prescribing guidelines. This trend persisted until early 2022, when the trend began to reverse. Whilst many complex factors influence prescribing behaviour and safety, area-level deprivation appears to be an important dimension.

## Introduction

Inhalers deliver medication directly to the lungs and are prescribed to treat respiratory conditions such as asthma and Chronic Obstructive Pulmonary Disease (COPD). Inhaled medication is directed to the airways providing either fast relief or long term control of symptoms. Upon asthma diagnosis, patients are usually first prescribed low-dose Inhaled Corticosteroids (ICS) as preventative or ‘maintenance’ inhalers. If these do not control symptoms or are not taken as advised, the ICS dose can be increased and additional inhalers such as long-acting beta agonists (LABA) and long-acting muscarinic antagonists (LAMA) can be prescribed. Patients can also be prescribed ‘rescue inhalers’ in the event of a flair up, or moderate on-going respiratory symptoms. Historically, and over the period examined in this study, this would have been short-acting beta agonists (SABA). Increasingly, however, ICS/LABA inhalers are recommended as a SABA-free approach, in line with the Maintenance and Reliever Therapy (MART) prescribing strategy^1^. ICS are also prescribed for COPD, though only in combination second- or third-line therapy, where LABA or LAMA constitute the first-line therapy. Although challenging to monitor due to varying treatment pathways, local administrative guidelines, and available products, monitoring how inhalers are prescribed is an important public health priority. It is estimated that problematic prescribing practices in the UK put 120,000 people at risk of potentially life-threatening asthma attacks^2^.

This study quantitatively profiles dispensed prescriptions of different inhaler categories at Lower Layer Super Output Area (LSOA) neighbourhood-level in England. It assesses prescribing behaviour by analysing categories of inhaler prescribing (Table 1) and generating guideline-based metrics (Table 2) derived from the National Institute for Health and Care Excellence (NICE)^3^ and the British Thoracic Society (BTS)^4^. These metrics reflect key contextual factors in prescribing, including disease severity, treatment norms, and practices that may, according to clinical guidelines, risk patient safety (e.g. through side effects, poor adherence, or suboptimal disease control) or contribute to environmental harm. Prescribing behaviour is explored in the context of neighbourhood-level deprivation, as measured by the Index of Multiple Deprivation (IMD)^5^. Analysing changes in prescribing behaviour at a period of unprecedented disruption — March 2020 and the onset of the COVID-19 pandemic — allows identification of areas where prescribing behaviour may deviate from the norm. From this, patterns of neighbourhood-level prescribing can be inferred pre- and post- disruption and by area-level deprivation.

**Table 1:** Inhaler subsets used in the analysis including derivations and contents.

Subset name	Contents	ICS	SABA	LABA	LAMA	Source
all_ics_products	All inhaled corticosteroids. Includes combination inhalers.	$\checkmark$		$\checkmark$	$\checkmark$	^6,7^
all_saba_ics_products	All inhaled corticosteroid inhalers and SABA inhalers. Includes combination inhalers.	$\checkmark$	$\checkmark$	$\checkmark$	$\checkmark$	^8^
high_dose_ics_products	All high dose inhaled corticosteroids as defined in Table 12 of the BTS/SIGN guidance^9^. Includes combination inhalers.	$\checkmark$		$\checkmark$	*	^6,7^
laba_products	LABA products. Includes combination inhalers.	$\checkmark$		$\checkmark$	$\checkmark$	^6^
lama_products	LAMA products. Includes combination inhalers.	$\checkmark$		$\checkmark$	$\checkmark$	^6^
mdi	All MDIs in BNF Chapter 3, excluding salbutamol.	$\checkmark$		$\checkmark$	$\checkmark$	^10^
mdi_dpi	All inhalers in BNF Chapter 3, excluding salbutamol.	$\checkmark$		$\checkmark$	$\checkmark$	^10^
saba_inhaler_products	Short acting beta agonist (SABA) inhalers — salbutamol and terbutaline, NOT including ICS.		$\checkmark$			^6^
**Derived subsets/other**						
dpi	mdi_dpi EXCLUDING mdi	$\checkmark$		$\checkmark$	$\checkmark$	$\wedge$
ics_laba	all_ics_products EXCLUDING (lama_products, ics_mono)	$\checkmark$		$\checkmark$		$\wedge$
ics_laba_lama_combo	all_ics_products EXCLUDING ics_mono	$\checkmark$		$\checkmark$	$\checkmark$	$\wedge$
ics_mono	all_ics_products EXCLUDING (laba_products, lama_products)	$\checkmark$				$\wedge$
laba_lama	(laba_products, lama_products) EXCLUDING all_ics_products			$\checkmark$	$\checkmark$	$\wedge$
patients_resp_lsoa	Number of asthma + COPD patients per LSOA by year.					^11^
salamol	BNF name includes “salamol”.		$\checkmark$			
ventolin	BNF name includes “ventolin”.		$\checkmark$			

*BNF* British National Formulary, *COPD* chronic obstructive pulmonary disease, *DPI* dry powder inhaler, *ICS* inhaled corticosteroids, *LABA* long-acting beta agonist, *LAMA* long-acting muscarinic antagonist, *LSOA* lower layer super output area, *MDI* metered-dose inhaler, *NHSBSA* national health service business service authority, *SABA* short-acting beta agonist. *May not have been excluded, but not present in subset, ⌃See above.

Prescribing data in this study were collected from OpenPrescribing.net^12^ and represent the number of dispensed prescription items. For every General Practice (GP) in England, the cost and quantity of prescriptions by drug, in this case inhaler type and brand, is published. GP practice data is attributed to LSOA spatial units via NHS Digital’s GP practice register datasets^13^, allowing GP-level prescribing to be approximated at neighbourhood-level. Seven prescribing measures are calculated for assessing inhaler and ICS prescribing (outlined in Table 2). These measures were chosen because they emphasise key factors in inhaler prescribing documented by NHS Business Services Authority (NHSBSA)^6^, or reveal interesting insights regarding neighbourhood-level deprivation and respiratory prescribing behaviours. After creating these measures, systematic variations are explored against neighbourhood-level deprivation and over time.

The paper presents a novel application of data from OpenPrescribing.net^12^. The aims were: 1) to use these data to profile neighbourhood-level prescribing patterns and examine their variation by area-level socioeconomic deprivation, given the established links between respiratory illness and deprivation; 2) to analyse changes in prescribing behaviour during a period of unprecedented disruption, and to assess whether, and how, normal prescribing activity subsequently resumed; and 3) to infer from the results how prescribing behaviours relate to deprivation and recommended practice. We provide insights and context that will be useful to health practitioners and policymakers interested in health inequalities and primary care service provision.

**Table 2:** Prescribing measures used in the analysis. Source and calculation from subsets.

Measure	Definition	Subsets for Calculation	Rationale	Reading	Source
*environmental inhalers* (Environmental measure).	MDIs dispensed as a % of all MDIs/DPIs dispensed.	(mdi/mdi_dpi) * 100	The NHS has committed to reducing its carbon footprint by 51% by 2025. DPIs emit less harmful greenhouse gas than traditional MDIs and the NHS long term plan supports the use of these inhalers where clinically appropriate.	Higher = ‘worse’.	^10^
*ics laba lama combo pppm* (’Severe’ asthma/COPD prescribing).	Number ICS/LABA/LAMA triple therapy inhaler prescriptions dispensed per asthma/COPD patient per month.	ics_laba_lama_combo/patients_resp_lsoa	ICS triple therapy is prescribed as regular maintenance for severe asthma and COPD. A relatively higher value between neighbourhoods would indicate greater prevalence of severe conditions compared to other severity levels.	Higher = ‘worse’.	^6^
*ics laba pppm* (’Moderate-Severe’ asthma/COPD prescribing).	Number ICS/LABA combination inhaler prescriptions dispensed per asthma/COPD patient per month.	ics_laba/patients_resp_lsoa	ICS/LABA combination inhalers are first line therapy for asthma, but only for COPD patients with high exacerbation risk. A relatively higher value between neighbourhoods would indicate greater prevalence of moderate-to-severe asthma cases compared to other severity levels.	Higher = ‘worse’.	^6^
*ics pppm* (’Mild-Moderate’ asthma prescribing).	Number ICS monotherapy inhaler prescriptions dispensed per asthma/COPD patient per month.	ics_mono/patients_resp_lsoa	ICS monotherapy inhalers are prescribed for mild-moderate asthma, but not recommended for COPD patients without asthma. A relatively higher value between neighbourhoods would indicate greater prevalence of mild-moderate asthma cases compared to other severity levels.	Lower = ‘worse’.	^6^
*ics dose* (High dose ICS).	Number ‘high dose’ ICS prescriptions dispensed as a % of all ICS-containing prescriptions.	(high_dose_ics_products/all_ics_products) * 100	Greater potential risk of systemic side effects with high-dose ICS products.	Higher = ‘worse’.	^6,7^
*laba ics* (LABA/LAMA to ICS-containing prescriptions ratio).	Number of LABA/LAMA mono/combination inhaler prescriptions dispensed for each ICS-containing prescription.	laba_lama/all_ics_products	LABA/LAMA monotherapies are never recommended as sole treatment for asthma, but are first-line treatments for COPD. A higher value in this measure could therefore indicate inappropriate prescribing for asthma, or treatment optimisation for COPD.	Higher = ‘worse’ (asthma)/Higher = ‘better’ (COPD).	GP advisor.
*saba* (Excess SABA prescribing).	SABA inhalers dispensed as a % of ICS and SABA inhalers.	(saba_inhaler_products/all_saba_ics_products) * 100	High use of SABA and poor adherence in the use of ICS in asthma suggests poor control. These patients should be reviewed regularly to ensure good control.	Higher = ‘worse’.	^8^

*COPD* chronic obstructive pulmonary disease, *DPI* dry powder inhaler, *GP* general practice, *ICS* inhaled corticosteroids, *LABA* long-acting beta agonist, *LAMA* long-acting muscarinic antagonists, *MDI* metered-dose inhaler, *SABA* short-acting beta agonist.

## Results

### Trends in number of prescriptions dispensed at national and neighbourhood-level

**Figure 1:**
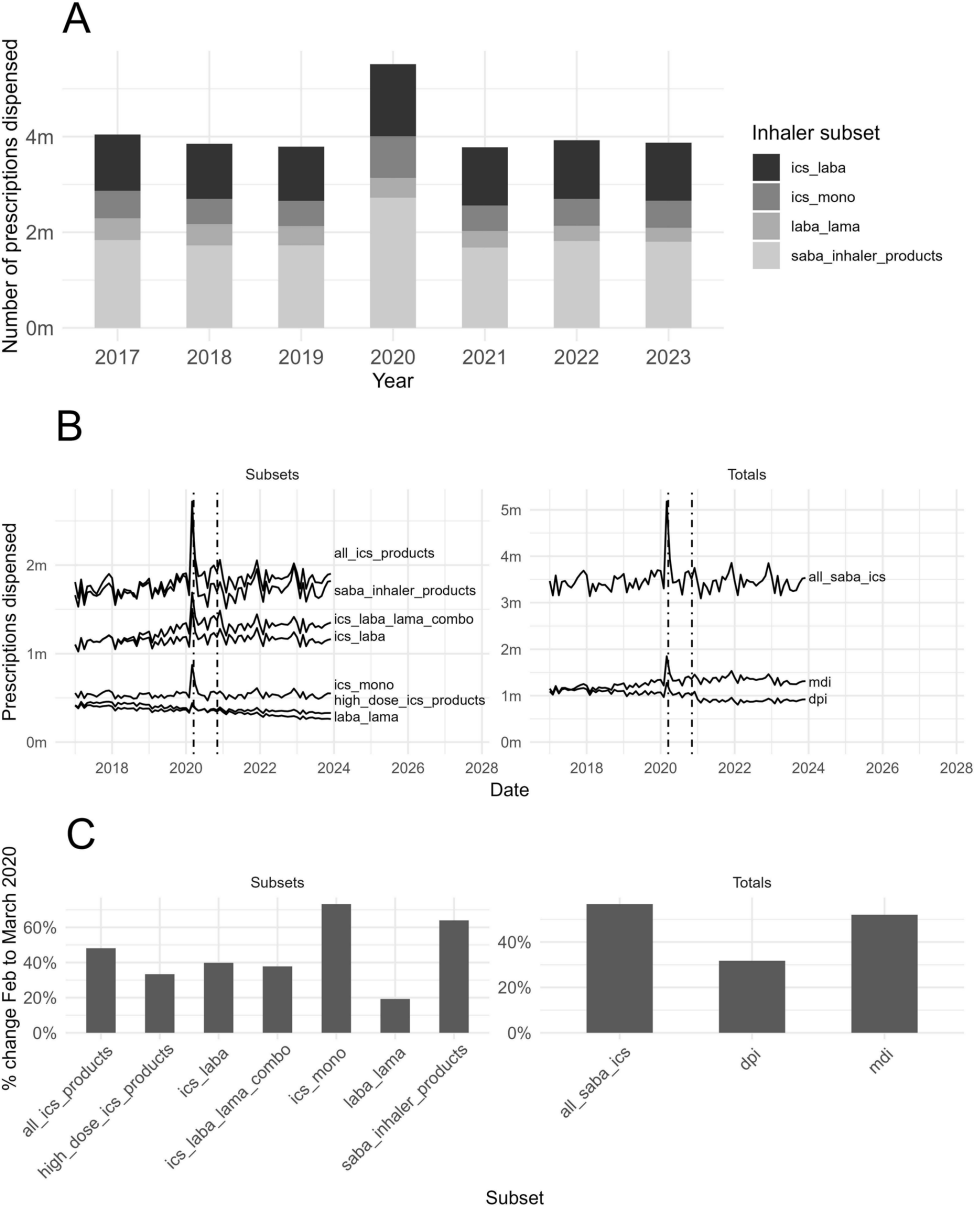
(**A**) Number of prescriptions dispensed in the month of March for England for the years 2017–2023 for key inhaler subsets. COVID-19 lockdown occurred in March 2020, resulting in extreme changes in prescribing behaviour. (**B**) Inhaler prescriptions dispensed in England month-by-month for the subsets in (**A**) along with some additional subsets, and national totals for all inhaler drugs and inhaler types. Dashed lines at national lockdowns. (**C**) Percentage increase in inhaler prescriptions dispensed in England from February to March 2020 for the categories displayed in (**B**) . *DPI* dry powder inhalers, *ICS* inhaled corticosteroid, *LABA* long-acting beta agonist, *LAMA* long-acting muscarinic antagonist, *MDI* metered-dose inhalers, *SABA* short-acting beta agonist.

We find marked and systematic differences in the number and type of inhalers prescribed pre- and post-March 2020 lockdown, and of outcomes across socio-economic groups. Figure 1 presents trends for prescription items dispensed in England by key inhaler subsets (defined in Table 1) for the years 2017–2023. Clearly observable is the increase in March 2020, coinciding with the onset of the COVID-19 lockdown. Overall a c. 50% increase is shown compared to the preceding and following three years (Fig. 1A), with ICS maintenance inhalers (monotherapy) showing the largest increase of c. 73% compared to February 2020 (Fig. 1C). The relative proportion of inhaler types dispensed was constant year-to-year, with SABA constituting approximately 50%, ICS monotherapy approximately 15%, ICS/LABA combination inhalers approximately 25%, and LABA/LAMA mono/combination inhalers approximately 10% of dispensed prescriptions. Month-on-month data show a sharp uptick in prescriptions dispensed in March 2020 as lockdown begins, which plateaus by April 2020, followed by a slight drop in August 2020 (Fig. 1B).

**Figure 2:**
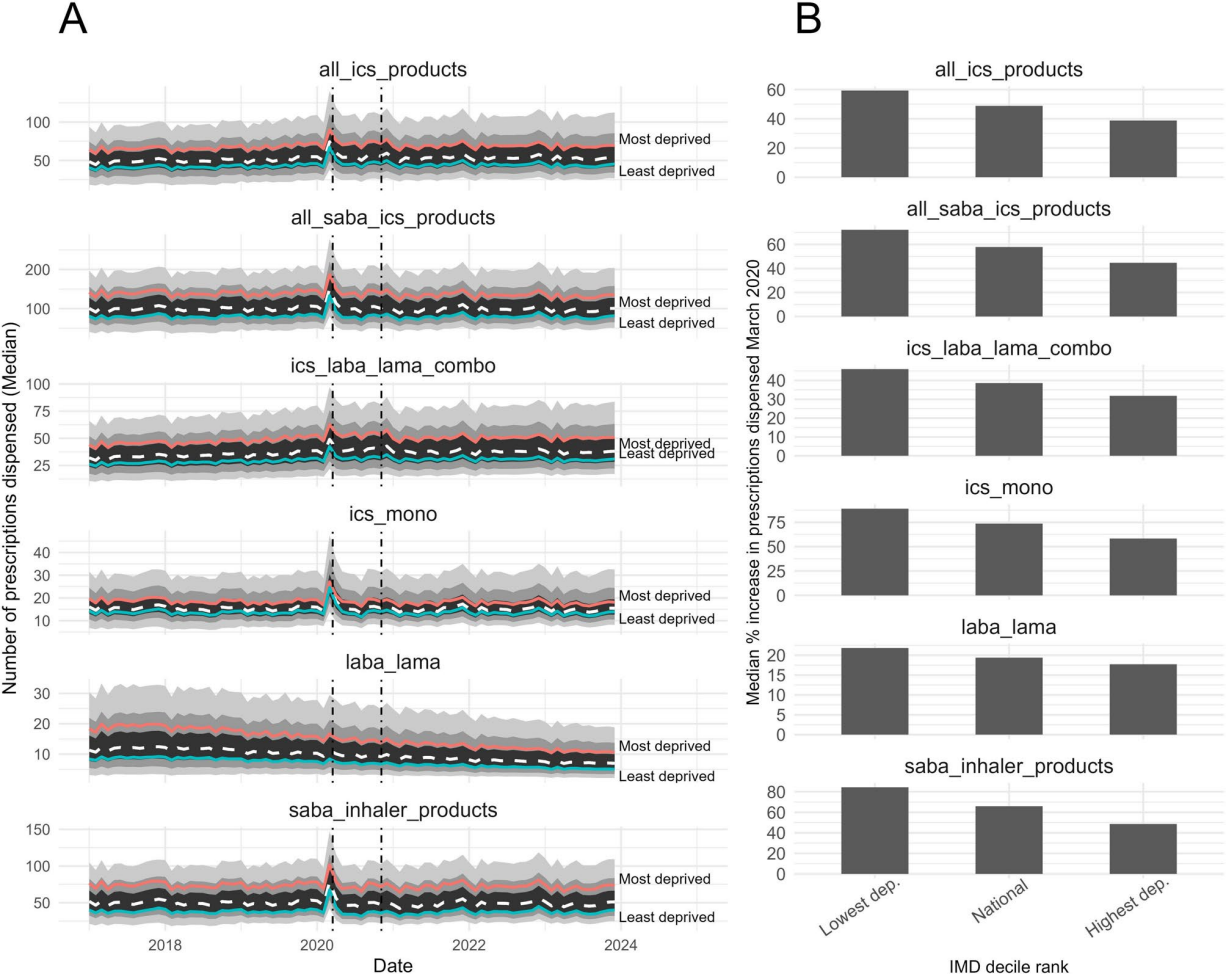
(**A**) Median number of prescriptions dispensed for different inhaler subsets at LSOA level. Dashed white lines show the national median, red and green lines show the median for LSOAs in the most and least deprived deciles respectively. The distribution of the sample is displayed with ribbons showing the 50th, 80th, and 95th percentile in black, dark grey, and light grey, respectively. (**B**) The median percentage increase in number of prescriptions dispensed for each subset for the LSOAs in the least deprived decile, the national median, and the LSOAs in the most deprived decile. *ICS* inhaled corticosteroid, *IMD* index of multiple deprivation, *LABA* long-acting beta agonist, *LAMA* long-acting muscarinic antagonist, *LSOA* lower layer super output area, *SABA* short-acting beta agonist.

In Fig. 2 are median average monthly prescription items dispensed at LSOA level nationally (dashed white lines) and for the most (red lines) and least (green lines) deprived deciles for key inhaler subsets. Regardless of type, more inhaler prescriptions are dispensed in the most deprived LSOAs compared to the least deprived, both at baseline pre-COVID-19 lockdown and the period following (Fig. 2A). However, for each subset the rate increase observed at lockdown was consistently lower in the most deprived decile than in the least deprived decile (Fig. 2B). The greater relative increase in the less deprived neighbourhoods is confirmed by correlation coefficients of counts and neighbourhood-level deprivation rank for all subsets except LABA/LAMA mono/combination inhalers.

### Descriptive analysis of prescribing measures

The seven prescribing measures in Table 2 and documented in the ‘Methods’ section aim to capture aspects of prescribing behaviour. Here we describe neighbourhood-level changes in each before, during, and after a time of intense and unprecedented disruption: the COVID-19 lockdown in March 2020.

**Figure 3:**
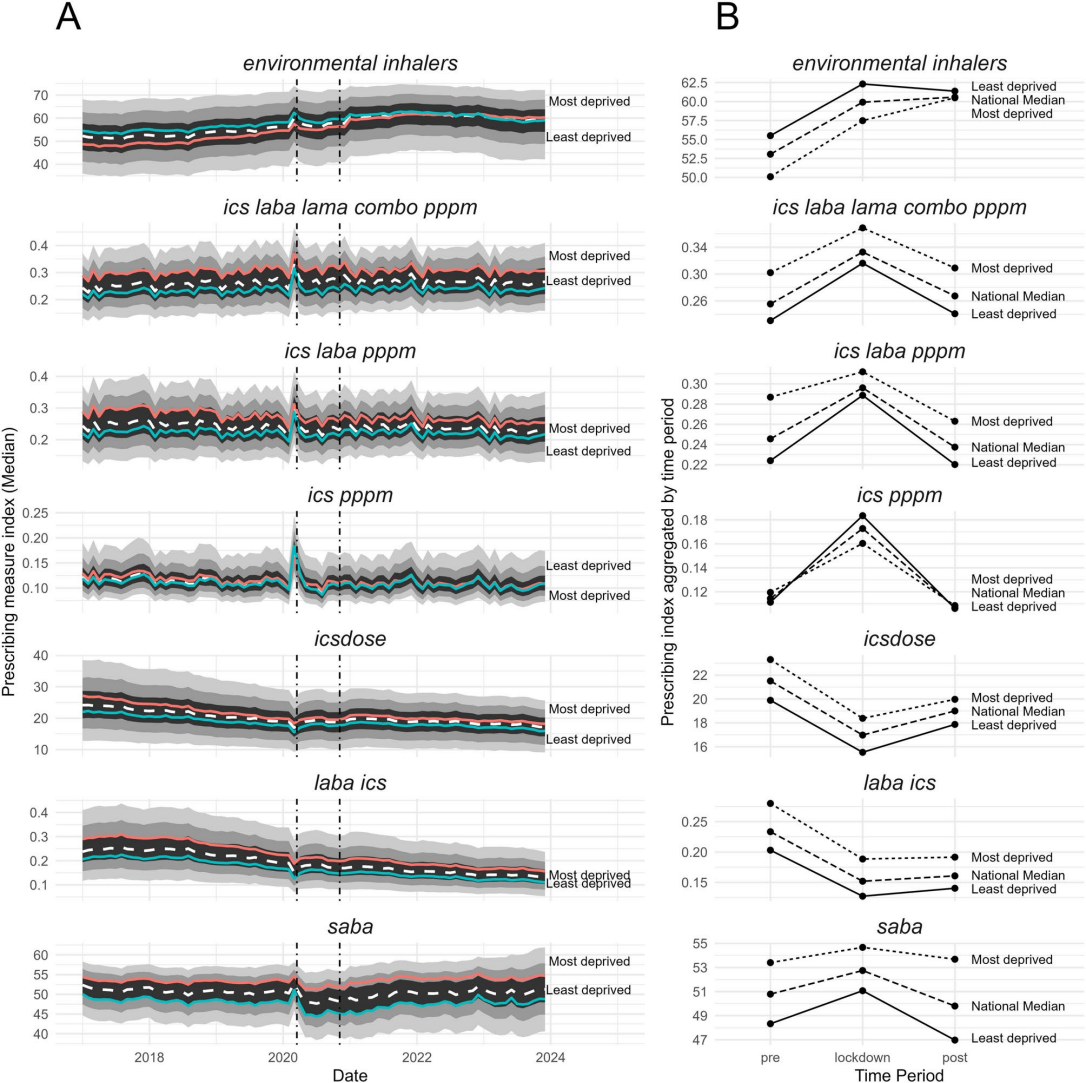
(**A**) Median values for prescribing measures at LSOA level. Dashed white lines show the national median, red and green lines show the median for LSOAs in the most and least deprived deciles, respectively. The distribution of the sample is displayed with ribbons showing the 50th, 80th, and 95th percentile in black, dark grey, and light grey, respectively. See Table 2 for interpretation. Vertical dashed lines indicate dates when national lockdowns were announced. (**B**) The median value for each prescribing measure aggregated to pre-lockdown, lockdown, and post-lockdown time periods for the LSOAs in the least deprived decile, the national median, and the LSOAs in the most deprived decile. Pre-lockdown includes all 38 months from January 2017–February 2020. Lockdown includes March 2020. Post-lockdown includes all 38 months from April 2020–May 2023. Data extend to December 2023, but we assess equal number of months pre/post. *combo* combination therapy, *ICS* inhaled corticosteroid, *pppm* per person per month, *LABA* long-acting beta agonist, *LAMA* long-acting muscarinic antagonist, *LSOA* lower layer super output area, *mono* monotherapy, *SABA* short-acting beta agonist.

#### Environmental impact of inhaler prescribing (*environmental inhalers*)

**Figure 4:**
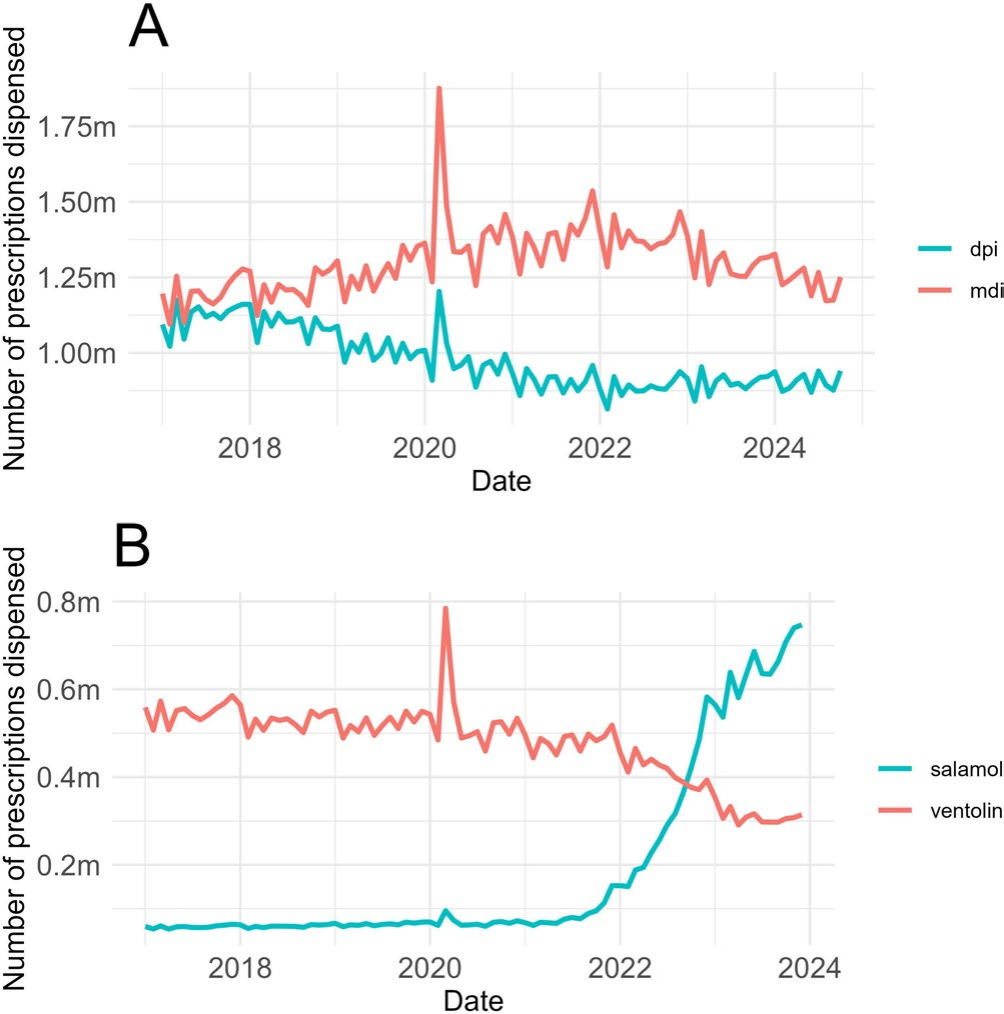
Environmental impact of inhaler prescribing. (**A**) Number of dry powder inhalers (DPIs) dispensed against metered-dose inhalers (MDIs) dispensed. DPIs are considered to be less harmful to the environment as they do not emit potent greenhouse gases (GHGs) (though it should be noted that DPIs have a larger environmental impact in other areas^14^). Shows divergence since around the start of 2018, followed by a trend towards convergence starting late 2021. (**B**) Number of inhalers prescribed for brands of inhalers with smaller (Salamol) and higher (Ventolin) carbon footprints^15^. Together, these inhalers account for around 50% of all SABA prescribing. *DPI* dry powder inhalers, *MDI* metered-dose inhalers, *NHS* National Health Service, *SABA* short-acting beta agonist.

Overall, the *environmental inhalers* measure — the number of metered dose inhalers (MDIs) dispensed as a percentage of all MDIs and dry powder inhalers (DPIs) — showed a persistent increase from the start of 2017 until around the end of 2021, when it plateaus before declining slightly. This indicates an increasing bias towards the prescribing of MDIs, until this trend reverses. There is a clear difference between the most and least deprived LSOAs pre-lockdown: the most deprived consistently dispense more DPIs (lower greenhouse gass emissions). Post-lockdown, this difference disappears. The total counts for these subsets illustrate the divergence in 2018 and the subsequent trend towards convergence in late 2021 at the national level (Fig. 4A). Since late 2021, we also see a drastic change in the prescriptions dispensed for specific brands of SABA inhalers known to have larger (Ventolin) and smaller (Salamol) carbon footprints (Fig. 4B). The sum of these two inhalers accounts for around 50% of all SABA prescribing.

**Figure 5:**
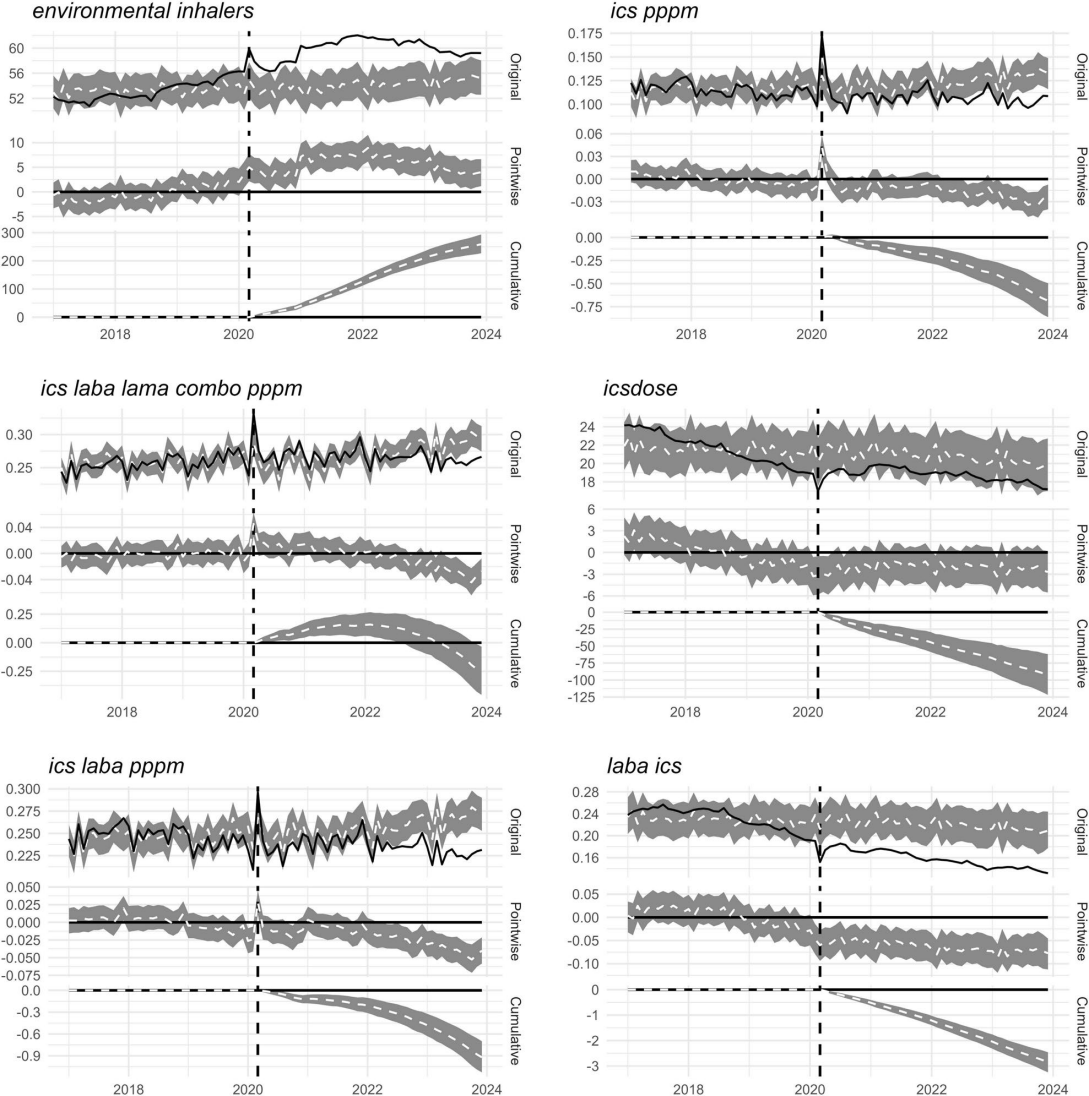
Interrupted Time Series (ITS) charts generated using the national median for each measure (see Fig. 6 for *saba*). Solid black lines show the observed data. Dashed white lines show a modelled *counterfactual* which represents expected trends given the pre-intervention period and a controlling covariate. The grey ribbon around the counterfactual represents a 95% Bayesian credible interval. Plots show the original data, and pointwise and cumulative subtractions between the observed and counterfactual data. Summary statistics for ITS analysis imply a difference between pre- and post- period for all data displayed (Bayesian one-sided tail-area probability p = 0.001). *combo* combination therapy, *ICS* inhaled corticosteroid, *pppm* per person per month, *LABA* long-acting beta agonist, *LAMA* long-acting muscarinic antagonist, *LSOA* lower layer super output area, *mono* monotherapy, *SABA* short-acting beta agonist.

#### Disease severity-related prescribing (*ics laba lama combo pppm*, *ics laba pppm*, and *ics pppm*)

The measures *ics pppm*, *ics laba pppm*, and *ics laba lama combo pppm* estimate numbers of inhaler prescriptions dispensed per patient per month for mild-moderate, moderate-severe, and severe respiratory conditions, respectively. Together, they are a useful proxy for assessing neighbourhood-level differences in the prevalence and severity of disease. Our results show that the number of prescriptions dispensed per patient for moderate-severe (*ics laba pppm*) and severe conditions (*ics laba lama combo pppm*) were similar between the national average and the least deprived neighbourhoods, but considerably higher in the most deprived neighbourhoods (Fig. 3). However, the number of prescriptions dispensed per patient for mild-moderate conditions (*ics pppm*) was comparable between the national median, and the most and least deprived neighbourhoods. All three displayed a transient increase at lockdown — the magnitude of change was largest for *ics pppm*, which is consistent with our finding that the ICS monotherapy subset showed the largest percentage increase of all those explored (Fig. 1C).

#### ICS side effect risk (*icsdose*)

We observed a higher proportion of high-dose ICS inhaler prescriptions dispensed in the most deprived neighbourhoods compared to the least deprived neighbourhoods (Fig. 3A, *icsdose*). Whilst this trend was sustained, the difference between the national average and the least and most deprived deciles was less than for other prescribing measures (Fig. 3A). This measure displayed a steady decrease until lockdown, where it showed decreased before stabilising at a level lower than before lockdown (Fig. 3B, *icsdose*).

#### LABA/LAMA to ICS-containing inhaler ratio (*laba ics*)

A higher value for the ratio of LABA/LAMA to all ICS-containing inhalers may indicate inappropriate prescribing for asthma, or potential treatment optimisation for COPD. This measure was higher in high-deprivation neighbourhoods than low-deprivation neighbourhoods, though both were within the 95th percentile (Fig. 3A, *laba ics*). The pre-lockdown period was higher compared to lockdown and post-lockdown values (Fig. 3B, *laba ics*).

#### Salbutamol over-prescribing (*saba*)

SABA dispensing as a proportion of all SABA/ICS inhalers (*saba*) was strongly associated with deprivation: it was higher in the most deprived IMD decile before, during, and after lockdown (Fig. 3A, *saba*). The magnitude of change in *saba* at lockdown was nevertheless smaller in high deprivation neighbourhoods than in low deprivation neighbourhoods (Fig. 3B). In the period following lockdown, *saba* reduced in both the most and least deprived deciles. However, the gap in *saba* between high and low deprivation neighbourhoods in the post-period widened (Fig. 3B).

### Estimating the impact of March 2020 on prescribing measures

Although the March 2020 lockdown radically disrupted the number of prescriptions dispensed, Figs. 1–3 demonstrate an apparent return to more ‘normal’ patterns of dispensing by April–May 2020. For some measures, however, there does appear to be a more systematic change in prescriptions dispensed, which can be difficult to infer via visual scanning of the figures. Interrupted Time Series (ITS) analysis allows us to identify whether prescribing levels of individual measures are significantly different in the post- period to that of the pre- period.

In Fig. 5 we present ITS charts displaying the observed data as a solid black line against a modelled *counterfactual* dashed white line. The counterfactual is arrived at using data on inhaler prescribing from the pre-intervention period and a controlling covariate — prescriptions dispensed across all drug codes over the entire period — which is assumed to be less directly impacted by the intervention (COVID-19 lockdown). The grey ribbon represents a 95% Bayesian credible interval (analogous to a frequentist confidence interval) to indicate the uncertainty around the estimated counterfactual. Plots show the original data, and pointwise and cumulative subtractions between the observed and counterfactual data.

**Figure 6:**
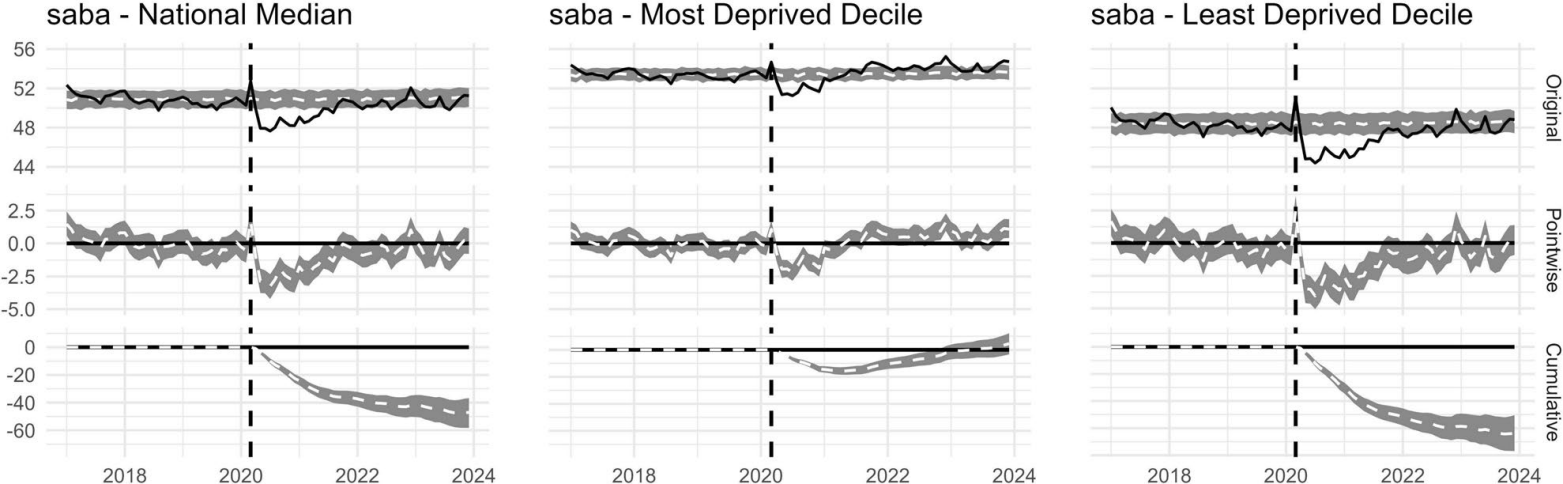
Interrupted Time Series (ITS) charts for the *saba* measure generated using the national median, and the median of LSOAs in the most and least deprived deciles. Solid black lines show the observed data. Dashed white lines show a modelled *counterfactual* which represents expected trends given the pre-intervention period and a controlling covariate. The grey ribbon represents a 95% Bayesian credible interval. Plots show the original data, and pointwise and cumulative subtractions between the observed and counterfactual data. Summary statistics for ITS analysis imply a difference between pre- and post- period for the national median and the median of LSOAs in the least deprived decile, but not for the most deprived (Bayesian one-sided tail-area probability p = 0.001). *LSOA* lower layer super output area, *SABA* short-acting beta agonist.

The summary statistics for the ITS analysis imply a difference between pre- and post- period data for all measures nationally, and for LSOAs in the highest and lowest deprivation deciles (Bayesian one-sided tail-area probability p = 0.001). The single exception to this rule was for *saba* in the highest deprivation LSOAs. For this reason, Fig. 5 presents the national median for all measures except *saba*, whilst Fig. 6 shows *saba* medians only for national, and highest and lowest deprivation deciles. The *icsdose* and *laba ics* measures show an observable decrease and *environmental inhalers* an observable increase in prescriptions dispensed over the study period. These patterns seem to be systematic trends that started long before March 2020. That the *counterfactual* plots for *environmental inhalers* and *icsdose*/*laba ics* model for increases and decreases in these measures further suggests this is a pattern not obviously attributable to March 2020.

## Discussion

By analysing patterns of prescriptions dispensed in England before, during, and after the March 2020 lockdown, our results confirm the onset of COVID-19 represented a moment of ‘global’ disruption in prescribing. More interesting are the differences we observe between small-area neighbourhoods. The primary aim of this paper is to demonstrate the novel use of data from OpenPrescribing.net^12^ for inferring differences in prescribing behaviour at the neighbourhood level. To this end, we noted that pre-pandemic, there were marked and systematic differences in the number and type of inhalers prescribed, and in prescribing measures in high and low deprivation neighbourhoods. Absolute counts of all inhaler types and proportions of rescue or emergency inhalers (SABA) were disproportionately high in the most deprived neighbourhoods. However, the largest increase during lockdown occurred in the least deprived LSOAs, with SABA and ICS monotherapy inhalers increasing by the highest percentage. These findings align with previous work^16^ which also reported a substantial increase in inhaler prescriptions in March 2020 and specifically for patients of higher socioeconomic status.

SABA inhalers, commonly referred to as ‘rescue’ inhalers^1^, were traditionally recommended for managing asthma or COPD flare-ups. However, recent guideline updates have reconsidered the clinical appropriateness of prescribing SABA for asthma, as excessive use has been linked to increased mortality risk^17^. Current best practice advocates for a SABA-free approach using ICS/LABA inhalers as rescue treatment instead^1^. Given these changes, we consider SABA over-prescribing a key indicator for concerning prescribing behaviour. That we observed an association between SABA overuse and neighbourhood-deprivation, a pattern reinforced in the months after lockdown, is therefore an important observation. It is consistent with earlier studies showing associations between SABA usage and income/employment deprivation^18^.

Regarding the environmental impact of inhalers, there is a trend in dispensing of less environmentally friendly MDI inhalers over DPI inhalers which persists until late 2021, whereby it reverses. DPIs emit less greenhouse gases than traditional MDIs and therefore can be considered more environmentally friendly^15^, although they have a larger environmental impact in other areas^14^. Tian et al. (2023) examined trends in relative MDI and DPI dispensing in detail^19^. Transitioning to low-carbon inhalers is a key strategy in the NHS’s efforts to reduce its carbon footprint^20^ and the use of these inhalers is encouraged where clinically appropriate. Prescribing suitability depends on such factors as the delivery mechanism and the amount of propellant, which varies by inhaler type. General practitioners may determine that switching inhalers could be detrimental to certain patients. For example, DPIs require sufficient inspiratory flow for effective medication delivery, which may pose a challenge for some patients and limit their suitability. We observed a worsening trend with the *environmental inhalers* measure until some time in late 2021 when it plateaued then showed an apparent reversal. The NHS Long Term Plan and SIGN158 asthma guidelines both support prescribing of DPIs over MDIs for environmental reasons where possible^9,21^. This reversal in prescribing trends most likely reflects the introduction of financial incentives for GPs to switch patients from MDIs to DPI, and from Ventolin to Salamol^22^. In December 2021, the introduction of this policy was delayed until April 2022.

Nationally, we find that the total number of dispensed inhalers, regardless of type, is higher in the most deprived areas compared to the least deprived. If based on increased need, this aligns with academic literature which reports that respiratory conditions and/or symptoms are more prevalent among lower socio-economic groups^23–25^. This is reflected in several of our measures, in particular those that capture prescriptions dispensed per patient in relation to disease severity (*ics laba lama combo pppm*, *ics laba pppm*, and *ics pppm*). We observed a higher number of inhalers dispensed per patient for moderate-severe and severe disease conditions in more deprived neighbourhoods, whilst drugs for mild-moderate conditions were prescribed at equal rates across levels of deprivation (though local policy and priorities may also contribute to variation in these measures). We also observed a consistently higher proportion of high-dose ICS inhalers being dispensed in more deprived areas with the *icsdose* measure, though the gap between the most and least deprived neighbourhoods was less pronounced than for other measures. Factors which exacerbate or cause respiratory conditions, and may explain our findings, include occupational exposure to hazardous agents^23^; worse air quality^26^; higher rates of smoking and exposure to second-hand smoke^24^; worse living conditions; and less-effective self-management of symptoms, also known as non-adherence.

Non-adherence may also explain higher rates of SABA (‘rescue’) inhaler dispensing in more deprived areas. If preventative inhalers are taken less frequently than prescribed, respiratory symptoms may be exacerbated and SABA ‘rescue’ inhalers would be required. Although the literature on non-adherence is not congruent^25^, many studies report that self-management of symptoms is worse among more deprived populations^24^, due to financial reasons (i.e., attempts to save on prescription costs) or accessibility-related issues in primary care. Longer wait times, lower GP practitioner to patient ratios, under-funding, and lack of physical access, all result in worse healthcare provision. The concept that healthcare resources are inversely correlated with area-level deprivation was originally coined as the ‘inverse care law’ by Tudor Hart in 1971^27^ but is shown to persist within NHS primary care today^28^.

Another key factor influencing GPs’ prescribing decisions is whether a patient has attended their annual review. When a patient requests additional inhalers, the system alerts the GP to check if the review has taken place. While GPs may withhold prescribing if the review has not been completed, they may also choose to prescribe regardless to avoid a potentially life-threatening situation. This factor may have contributed to the differences observed in the *laba ics* measure before and after the COVID-19 lockdown. Prior to lockdown, this measure was higher and more variable, whereas post-lockdown, both the median value and variability decreased. We speculate that large numbers of non-adherent patients undergoing treatment reviews and subsequent optimisation during lockdown could result in lasting effects on prescribing patterns.

All neighbourhoods experienced altered asthma- and COPD- prescribing behaviour during the COVID-19 lockdown, but the most deprived neighbourhoods exhibited both higher quantities of prescriptions dispensed, and changes in prescribing measures, before, during, and after that unprecedented period of disruption. Explanatory factors could include higher incidence of respiratory conditions, less effective symptom management, or more non-adherence in more deprived neighbourhoods. Small area-level insights not only allow for a better understanding of systematic differences in local prescribing within the health system, but they can also uncover geographic variations in socio-economic and environmental causes of respiratory diseases. This can complement the patient-centred clinical approach and provide a pathway for more broad-scale interventions.

Our prescribing measures can be used to analyse national prescribing trends over time, identify potential issues within local health systems, and highlight populations at risk- whether from negative effects of prescribing behaviour, or from increased likelihood of more severe respiratory diseases and outcomes. More research is needed to untangle the complex relationship between prescribing behaviour and area-level characteristics. Local prescribing patterns may be influenced by the socio-economic and environmental conditions of an area and its population, but also by the structure and governance of the local healthcare system itself. Integrated Care Boards (ICBs), which oversee healthcare provision at the regional level, vary in their governance frameworks, financial resources, and strategic priorities. These differences can directly impact prescribing practices, healthcare access, and the support available for managing respiratory conditions. Consequently, certain populations may be at greater risk of experiencing negative effects of prescribing behaviour or more severe respiratory outcomes based on the healthcare system they rely on. Understanding these dynamics requires further investigation to ensure equitable and effective respiratory care across different healthcare settings.

One possible approach to better understand the relationship between prescribing behaviour, area-level characteristics, and local healthcare system factors is through the use of linked data. By integrating prescribing records with other datasets — such as patient demographics, healthcare utilisation, environmental exposures, and socio-economic indicators — it becomes possible to build a more comprehensive picture of the drivers of prescribing variability. Linking prescribing data with hospital admissions, primary care records, and outcomes data could help identify whether certain prescribing patterns contribute to better or worse patient outcomes and whether specific populations are disproportionately affected by deviations in prescribing behaviour. The novel use of data from OpenPrescribing.net^12^ for analysing neighbourhood-level prescribing demonstrated in this paper requires validation in future studies, which could be achieved through the use of primary care records.

## Methods

### Data

#### Dispensing data

OpenPrescribing.net^12^ is an online portal through which anonymised prescribing data published by NHS Digital can be conveniently accessed. Its application programming interface (API) enables GP practice IDs to be retrieved, and from there, searches constrained by GP practice, British National Formulary (BNF) codes, and temporal extent. From the API, prescription cost and number of prescription items dispensed monthly were collected for all inhaler types and brands at practice-level. Additionally, details on GP practices, their corresponding Sub-Integrated Care Board (Sub-ICB) codes, practice names, postcode, and practice type were obtained. Since the data published by OpenPrescribing.net^12^ constitutes the number of prescriptions dispensed, rather the number written, we refer to these data as ‘dispensing data’ where appropriate.

#### Population data

Number of patients registered at GP practices, Sub-Integrated Care Board Location (SICBL), and other administrative geographies were obtained from https://digital.nhs.uk^13^. This included a dataset recording the number of patients registered at each LSOA for each GP practice. Annual numbers of patients registered by GP practice who had been diagnosed with asthma or COPD, as well as the number patients having attended their annual review, were obtained from the NHS Digital Quality Outcomes Framework (QOF)^11^. Data for linking GP practice postcodes to LSOA and SICBL codes and boundaries were downloaded from the Office for National Statistics (ONS) Geography Portal^29^, and Index of Multiple Deprivation 2019 data from the ONS Census Data Service^30^.

### Inhaler subsets

The dispensing data retrieved from OpenPrescribing.net^12^ were sourced using BNF drug codes for individual inhaler products. The specific BNF codes recovered came from drug lists on the NHSBSA Respiratory Dashboard^6^ (see the specification document for comparators on which some of our measures were based and Appendix 2 for drug codes) and from measures provided by OpenPrescribing.net^7,8,10^. We used these drug lists to create specific inhaler subsets to perform descriptive data analysis of the number of prescriptions dispensed, and to calculate prescribing measures described in the next section. Lists of BNF codes were combined, taken as provided, or adapted to generate derivative subsets, described in Table 1. The subsets are assigned names which are referenced in subsequent figures to aid interpretation.

### Measures

The measures on which our analysis is based are defined in Table 2, along with the names of the inhaler subsets used to calculate them. Each measure uses the total number of monthly items dispensed for the stated inhaler type, recorded at GP-practice level, as well as information on the patient register, describing for instance, numbers of patients listed with asthma and COPD.

The rationale for each of the measures was derived from NHS Business Services Authority (NHSBSA) guidelines^6^ and via consultation with an experienced NHS General Practitioner working in the UK. We create an *environmental inhalers* measure, expressed as the relative share of metered-dose inhalers (MDI) to dry-powder inhalers (DPIs), which do not emit potent greenhouse gases (GHGs)^31^, though on other criteria do have negative environmental impacts^14^. *ics laba lama combo pppm*, *ics laba pppm*, and *ics pppm* describe the respective number of preventer, maintenance, and triple therapy inhalers dispensed monthly per COPD/asthma patient. These three measures incorporate patient counts recorded at LSOA level with asthma and COPD. They provide approximate estimates of adherence to prescriptions written for increasing levels of disease severity (albeit limited within this analysis by our not having linked prescription data). The measure *ics dose* was created as increased side-effects and poorer overall outcomes have been self-reported^32^ and observed^33^ with high-dose ICS inhalers, as compared to low-dose inhalers. The *laba ics* measure captures prescribing behaviour related to LABA/LAMA mono/combination therapy relative to all ICS-containing inhalers. For asthma, LABA/LAMA monotherapy is never a recommended prescription, and only in combination therapy as an add-on. However, for COPD, both LABA and LAMA are first-line therapies. Since we cannot separate prescriptions written for asthma and COPD, we cannot directly discriminate between prescribing that is dangerous for asthma from that which is a treatment optimisation for COPD. However, this measure holds some value for exploring asthma/COPD prevalence and treatment adherence patterns between neighbourhoods of different deprivation-level. A more straightforward case of concerning prescribing behaviour or poor management of existing symptoms, is where ‘rescue’ inhalers are over-prescribed. The final measure *saba* therefore calculates the share of prescriptions dispensed for rescue inhalers relative to all ICS/SABA-containing maintainer inhalers.

To estimate prescribing at the neighbourhood-level (LSOA), we used NHS Digital’s openly published GP register datasets^13^. For each GP practice, the number of patients in each LSOA registered at the practice is provided. We assume that there are no systematic differences in the types of patient living in the LSOAs linked to a particular practice, and so allocate the GP-practice prescription items dispensed to an LSOA according to the proportion of patients each LSOA contributes. So if an LSOA contributes 20% of a GP’s overall patient numbers and the practice dispenses 100 ICS items in March 2020, then the LSOA receives an item count for ICS of 20 from that practice.

We conducted a temporal analysis to explore average values for prescribing measures at the March 2020 lockdown compared to aggregated periods before and after. The pre-lockdown period included 38 months from January 2017–February 2020, the post-lockdown period included 38 months from April 2020–May 2023, and lockdown constituted the single month of March 2020. We had access to data extending to December 2023, but limiting the aggregated analysis to May 2023 allowed us to include an equal number of months pre- and post- for a fairer comparison. Averages (median) values were calculated by IMD decile, paying particular attention to differences between the 10% most deprived LSOAs, the 10% least deprived LSOAs, and the national average.

In order to compare more formally changes in prescriptions dispensed in the pre- and post- March 2020 periods, we implement an interrupted time series analysis (ITS)^34^. Expected prescribing levels are modelled using the temporal prescribing pattern in the pre-period as a covariate, along with the total number of prescriptions dispensed across all drug codes during both the pre- and post-intervention periods (the *control*). The control data are collected from NHS Open Data Portal. Given that COVID-19 is a respiratory virus, our assumption is that *inhaler* prescribing was acutely affected by the intervention (March 2020) and so we regard global prescribing to be a reasonable controlling comparator. Analysing the cumulative difference between observed prescribing against this expectation allows us to infer the impact of March 2020 on dispensed prescriptions against the counterfactual that *inhaler* prescriptions were not specially affected.

### Limitations

The necessary ethical requirement that OpenPrescribing.net^12^ does not publish linked prescription records for individual patients limits the richness with which we can profile prescribing behaviour and judge symptom management. For example, it would be instructive to explore individual-level prescribing trajectories: the circumstances under which prescriptions switch from low-dose ICS to high-dose ICS in order to manage symptoms, the extent to which rescue inhalers are prescribed in isolation or in addition to maintenance inhalers, and whether there is a neighbourhood-level dimension to this. Data from OpenPrescribing.net^12^ does not reflect the number of prescriptions written, but the number of prescriptions dispensed. This may have had some deprivation-dependent effects, such as avoidance of prescription charges, that we were unable to account for in this study. Additionally, whilst the frequency of a drug being prescribed is known, we do not know the quantity with which it is prescribed — e.g., the number of inhalers provided in each prescription. Finally, the conversion from GP surgery-level to LSOA-level data provides a weighted estimate of dispensing data, assuming that prescriptions are dispensed in proportion to the number of registered patients in that LSOA. In reality dispensed prescriptions will not be distributed uniformly across registered patients and so our neighbourhood-level measures are subject to uncertainty.

### Reproducibility and code

Replication materials, with code for collecting and processing data and reproducing the analysis, is via the paper’s accompanying code repository. A directory containing datasets harvested from OpenPrescribing.net^12^ can be provided by the authors on request.

## Data Availability

Data collection code and analysis materials are available via the project’s github repository. A folder containing harvested data can be provided by the corresponding author on request.
